# Grafting Hyperbranched Polymers onto TiO_2_ Nanoparticles via Thiol-yne Click Chemistry and Its Effect on the Mechanical, Thermal and Surface Properties of Polyurethane Coating

**DOI:** 10.3390/ma12172817

**Published:** 2019-09-02

**Authors:** Feng Zhan, Lei Xiong, Fang Liu, Chenying Li

**Affiliations:** School of Materials Science and Engineering, Nanchang Hangkong University, Nanchang 330063, China

**Keywords:** TiO_2_, hyperbranched polymers, polyurethane, nanocomposite coatings, thiol-yne click chemistry, mechanical and thermal properties, superhydrophobic property

## Abstract

In this study, we proposed a novel and facile method to modify the surface of TiO_2_ nanoparticles and investigated the influence of the surface-modified TiO_2_ nanoparticles as an additive in a polyurethane (PU) coating. The hyperbranched polymers (HBP) were grafted on the surface of TiO_2_ nanoparticles via the thiol-yne click chemistry to reduce the aggregation of nanoparticles and increase the interaction between TiO_2_ and polymer matrices. The grafting of HBP on the TiO_2_ nanoparticles surface was investigated by means of X-ray photoelectron spectroscopy (XPS), X-ray diffraction (XRD), Fourier transform infrared (FT-IR), nuclear magnetic resonance (NMR) and thermogravimetry analysis (TGA). The thermal and mechanical properties of nanocomposite coatings containing various amounts of TiO_2_ nanoparticles were measured by dynamic mechanical thermal (DMTA) and tensile strength measurement. Moreover, the surface structure and properties of the newly prepared nanocomposite coatings were examined. The experimental results demonstrate that the incorporation of the surface-modified TiO_2_ nanoparticles can improve the mechanical and thermal properties of nanocomposite coatings. The results also reveal that the surface modification of TiO_2_ with the HBP chains improves the nanoparticle dispersion, and the coating surface shows a lotus leaf-like microstructure. Thus, the functional nanocomposite coatings exhibit superhydrophobic properties, good photocatalytic depollution performance, and high stripping resistance.

## 1. Introduction

Titanium dioxide (TiO_2_) nanoparticles have been initially applied in the fields of photoelectrochemical solar cell and environmental photocatalysis, because of their strong oxidation and reduction ability [1,2,3,4]. Most recently, the application of TiO_2_-based photocatalytic products in superhydrophobic coatings is attracting considerable attention. Based on the photocatalytic and superhydrophobic properties of TiO_2_, the incorporation of TiO_2_ into coatings can increase the superhydrophobicity of coatings and keep the superhydrophobic surface clean by the action of sunlight and rainwater [5,6,7]. The contaminants, such as dust, can be easily removed by rainwater due to the superhydrophobic property, while the organic contaminants adsorbed on the superhydrophobic coatings containing TiO_2_ nanoparticles can be gradually decomposed by the photocatalytic action of TiO_2_ nanoparticles [8,9,10,11,12].

However, to obtain the desirable surface and mechanical properties of nanocomposite coatings, it is necessary to make the TiO_2_ nanoparticles disperse uniformly in the polymer matrix [13], although TiO_2_ nanoparticles tend to aggregate due to their extremely large specific surface area, which can affect their photocatalytic efficiency and functionality [14,15,16,17]. As a result, a great deal of efforts has been made to overcome this problem and increase the interaction between TiO_2_ and the matrix [18]. The aggregation of nanoparticles in the polymer matrix can be minimized by grafting the polymer chains onto the nanoparticles surface. The introduction of polymer chains on TiO_2_ nanoparticles not only improves their dispersion and compatibility with the polymer matrix, but also facilitates the formation of chemical or physical interaction between nanoparticles and the polymer matrix [19,20,21,22].

The introduction of polymer chains onto the TiO_2_ nanoparticle surface can be achieved by many different techniques, such as radical polymerization, ring-opening polymerization (ROP), reversible addition-fragmentation transfer (RAFT), and atom transfer radical polymerization (ATRP) [23,24,25,26]. Chen et al. [27] prepared a superhydrophobic material by grafting polystyrene onto a TiO_2_ surface via an in situ free-radical polymerization strategy. The coating containing the modified TiO_2_ showed a strong adhesive property, and the water contact angle (CA) was measured at 153.5 ± 1.5°, suggesting that this coating possessed superhydrophobic properties. Vergnat et al. [28] designed a robust approach to graft polymer brushes onto TiO_2_ nanoparticles via ATRP. The grafting of polymer brushes could enhance the dispersion of TiO_2_ nanoparticles and interaction between TiO_2_ and the polymer matrix, resulting in the significant improvement of the mechanical properties of nanocomposites.

However, most of these methods are difficult to apply because of their complex and time-consuming process, environmental pollution, and the requirement of specialized equipment. Recently, much attention has been paid to the grafting method based on click chemistry. Compared with the traditional surface grafting methods, this method exhibits many advantages, including a high selectivity and reaction speed, gentle experimental conditions, a high conversion efficiency, and its environmentally friendly nature, which has been used to graft polymer chains onto inorganic materials [29,30,31].

In this work, the hyperbranched polymers (HBP) were grafted onto the TiO_2_ surface via thiol-yne click chemistry. The introduction of HBP onto a TiO_2_ surface can introduce a large number of functional groups on the surface and greatly reduce the tendency of aggregation of TiO_2_ nanoparticles, resulting in the improvement of dispersion and interaction between TiO_2_ and the matrix [32,33]. According to our knowledge, few studies have been reported on the grafting of hyperbranched polymers onto TiO_2_ nanoparticles by this method. The grafting of HBP onto the TiO_2_ nanoparticle surface was characterized with X-ray photoelectron spectroscopy (XPS), X-ray diffraction (XRD), Fourier transform infrared (FT-IR), nuclear magnetic resonance (NMR) and thermogravimetry analysis (TGA) techniques. The influence of TiO_2_ nanoparticles grafted with HBP on the mechanical, thermal, and surface properties of polyurethane-based nanocomposite coatings was also extensively investigated.

## 2. Experimental

### 2.1. Materials

TiO_2_ nanoparticles with an average particle size of 50 ± 10 nm were purchased from Zhoushan Nano-materials Co., Ltd (Zhoushan, Zhejiang, China). (3-Mercaptopropyl) trimethoxysilane (KH590) was obtained from Aladdin. 2, 2-Dimethoxy-2-phenylacetophenone (DMPA) was purchased from Sigma-Aldrich Corporation (St. Louis, MO, USA) and used as a UV photoinitiator. The commercial polyurethane suspension (PU116) was obtained from Hefei Anke Fine Chemicals Co., Ltd (Hehei, China). All other reagents used in this paper were commercially available and used without further purification.

### 2.2. Synthesis of But-3-ynyl 3-Mercaptopropanoate

The hyperbranched monomer but-3-ynyl 3-mercaptopropanoate (BYMP) was synthesized according to the procedures described in the literature [34]. Typically, 12.5 g of but-3-yn-1-ol, 3.85 g of 3-mercaptopropionic acid, and 1.06 g of sulphuric acid were added to a flask, and the mixture was then stirred at 85 °C for 2 h. After reaction, the mixture was cooled to room temperature and dissolved in dichloromethane. The triethylamine was then added into the mixture, followed by washing with distilled water several times. The crude product was finally purified by silica gel chromatography using an eluent of hexane/ethyl acetate (4:1) to obtain pure BYMP. The chemical structure of BYMP was confirmed by ^1^H NMR. ^1^H NMR (600 MHz, CDCl_3_, δ/ppm): 2.5 (1H, H–C≡C), 2.1 (2H, C≡C–CH_2_–CH_2_–O), 1.7 (1H, SH), 4.5 (2H, C≡C–CH_2_–CH_2_–O), and 2.8 (4H, C(O)–CH_2_–CH_2_–S).

### 2.3. Immobilization of KH590 onto TiO_2_ Nanoparticles

Specifically, as-received TiO_2_ nanoparticles (500 mg) were initially dispersed into 100 mL toluene under ultrasonic agitation for 2 h. Then 100 mg of KH590 was added into the TiO_2_ dispersion, followed by 2 h of ultrasonication. The reaction mixture was stirred at 115 °C for 4 h under reflux and then filtered by air pump filtration. The filtered mass was purified by repeated washing with toluene, and was vacuum-dried at 100 °C for 48 h. The resulted products were noted as KH590 functionalized TiO_2_ (TiO_2_-SH).

### 2.4. Preparation of TiO_2_ Nanoparticles Grafted with Hyperbranched Polymers

**Figure 1:**
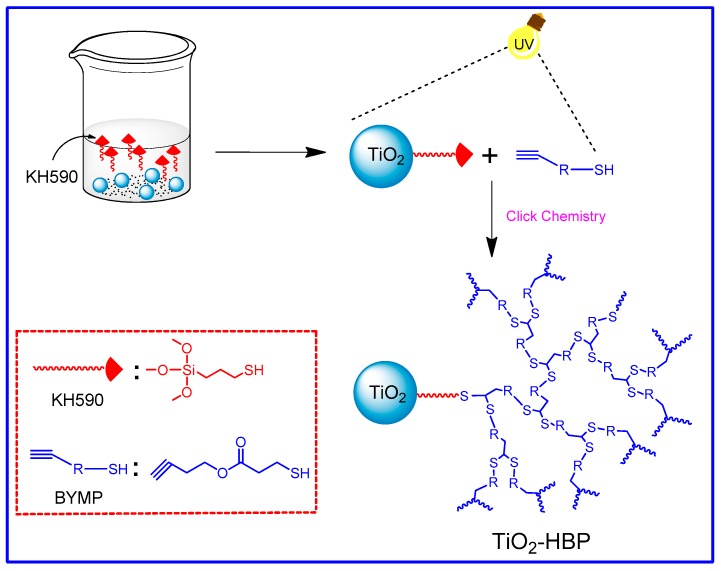
Synthetic route of TiO_2_ nanoparticles grafted with hyperbranched polymers (TiO_2_-HBP).

In a typical experiment, 0.5 g of TiO_2_-SH, 0.8 g of hyperbranched monomer BYMP, and 20 mL of dimethyl formamide (DMF) were added into a beaker, followed by 2 h of ultrasonication. Then an appropriate amount of photoinitiator DMPA was added into the beaker. The beaker was irradiated for 30 min under a 365 nm UV light at room temperature to perform the thiol-yne click reaction between the TiO_2_-SH and the hyperbranched monomers. After reaction, the mixture was washed with dichloromethane and Soxhlet extracted for 24 h to remove the physically adsorbed polymers and unreacted monomers. The resulted products were vacuum dried at 80 °C for 24 h to obtain TiO_2_ nanoparticles grafted with hyperbranched polymers (TiO_2_-HBP). The whole preparation process is shown in Figure 1.

### 2.5. Cleaving Hyperbranched Polymers from TiO_2_-HBP

In this work, the hyperbranched polymers were cleaved from the TiO_2_-HBP surface to determine the structure of grafted polymers. Typically, 200 mg of TiO_2_-HBP was immersed in 100 mL of 2 M HCl, and the solution was stirred under reflux for 24 h. The solution was then neutralized with NaHCO_3_, washed with abundant dichloromethane, and vacuum-filtered to remove the TiO_2_ nanoparticles. The resulted hyperbranched polymer was dried under vacuum at 70 °C for 24 h and subjected to NMR analyses.

### 2.6. Preparation of TiO_2_/Polyurethane Nanocomposite Coatings

The raw TiO_2_ or TiO_2_-HBP was first dispersed in tetrahydrofuran (THF) and sonicated for 2 h to prepare TiO_2_ suspension. Then, the TiO_2_ suspension was slowly added into the PU suspension and stirred at 4000 rpm for 2 h, followed by degassing in vacuum for 30 min. The TiO_2_/PU nanocomposite coatings were prepared on the glass substrate with a wet film thickness of 100–150 μm by using a film applicator. The free-standing films with a wet thickness of 200–250 μm were obtained by applying the TiO_2_/PU nanocomposite coatings to the polystyrene sheets. The resulted films were cured at room temperature for one week. The composition of nanocomposite coatings in this work was denoted as raw TiO_2_/PU-X or TiO_2_-HBP/PU-X, where X denoted as the content of nanoparticles, and the content of nanoparticles in the nanocomposite coatings was varied as 0, 2, 4, 6 and 8 wt%, respectively.

### 2.7. Characterization

Fourier transform infrared (FT-IR) measurements were carried on a Shimadzu IR Prestige-21 spectrometer in the wavenumber range of 500–4000 cm^−1^. The surface composition of nanoparticles was obtained from AXIS X-ray photoelectron spectra. The scanning electron microscopy (SEM) images were obtained from Quanta 200 F field emission SEM system (Eindhoven, Netherlands). X-ray diffraction (XRD) patterns of samples were obtained from a Bruker D8 X-ray diffractometer (Karlsruhe, Germany). The water contact angle (CA) measurement was performed on a JC2000C contact angle tester (Shanghai, China) at room temperature. The water CA values were measured at five different areas on each sample, and the average value was calculated. Thermogravimetric analysis (TGA) was performed on a TGAQ50 instrument (New Castle, DE, USA). The nuclear magnetic resonance (NMR) spectra were acquired using a Bruker DMX 600 spectrometer (Karlsruhe, Germany), employing TMS as an internal standard. The dynamic mechanical thermal analysis (DMTA) was carried out on a TA Q800-RSA3 dynamic mechanical analyzer (New Castle, DE, USA) under N_2_ atmosphere. Tensile strength and elongation at break of free-standing films were tested on a universal testing machine following the ASTM D2370 standard [35]. The tensile test was performed at 5 mm/min cross-head speed, and more than seven specimens were tested for each sample.

## 3. Results and Discussion

### 3.1. Characterization of TiO_2_-HBP

**Figure 2:**
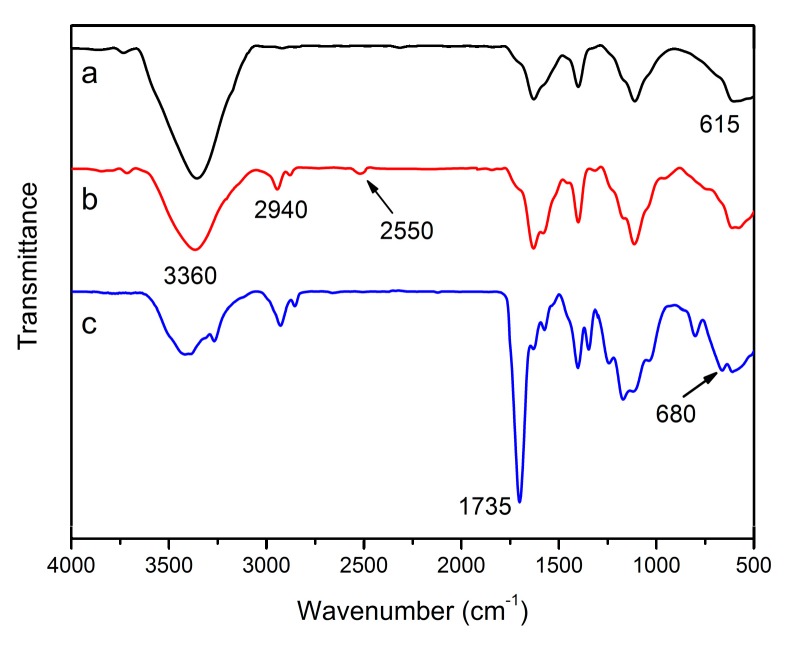
Fourier transform infrared (FT-IR) spectra of (**a**) raw TiO_2_, (**b**) TiO_2_-KH590 and (**c**) TiO_2_- hyperbranched polymers (HBPs).

FT-IR measurements were adequately performed to observe the chemical structure of the prepared TiO_2_ after each stage of modification. As shown in Figure 2a, FT-IR spectrum of raw TiO_2_ nanoparticles exhibits a strong absorption peak at 3360 cm^−1^ which can be assigned to –OH groups. Ti-O bands at 615 cm^−1^ can also be observed in this spectrum. From the FT-IR spectrum of TiO_2_-KH590 in Figure 2b, the absorption peak ascribed to –OH groups became weaker after the immobilization of KH590 on TiO_2_ nanoparticles, which is due to the hydrolytic condensation between the –OH groups on the surface of raw TiO_2_ nanoparticles and KH590. In addition, the FT-IR spectrum of TiO_2_-KH590 exhibits new peaks at 2940 cm^−1^ and 2550 cm^−1^ assigned to –CH_2_ and –SH groups of the grafted KH590. Compared to the TiO_2_-KH590 spectrum, the appearance of the vibration of C–S–C at 680 cm^−1^ and the decrease of the –SH absorption at 2550 cm^−1^ in TiO_2_-HBP spectrum (Figure 2c) implies that the thiol-yne click reaction has occurred between the –SH groups on the TiO_2_ surface and hyperbranched monomer. Meanwhile, the characteristic peaks of –CH_2_ at 2940 cm^−1^ and C=O at 1735 cm^−1^ have been distinctly enhanced due to the grafting of hyperbranched polymers. It is concluded that the hyperbranched polymers were successfully grafted onto the TiO_2_ surface via thiol-yne click reaction.

**Figure 3:**
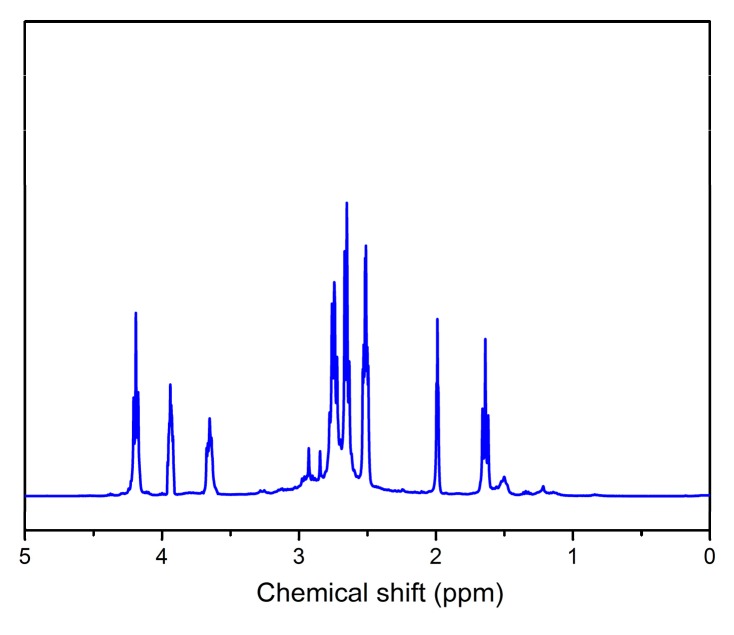
^1^H nuclear magnetic resonance (NMR) spectrum of HBP cleaved from the TiO_2_-HBP surface.

^1^H NMR spectroscopy can also prove the success of thiol-yne click reaction and the structure of hyperbranched polymers grafted on TiO_2_ surface. Figure 3 shows the ^1^H NMR spectrum of hyperbranched polymers cleaved from TiO_2_-HBP. The proton peak at 3.9 ppm is ascribed to the ester linkage after click reaction of the alkyne to form a saturated bond (O–CH_2_–CH_2_–CH_2_–C). The signals at 2.5 and 4.2 ppm can be assigned to the unreacted alkyne proton (–C**≡**CH) and ester linkage near the alkyne (O–CH_2_–CH_2_–C**≡**C), respectively, indicating that the resulted hyperbranched polymer still has alkyne end groups. In addition, a peak at 3.7 ppm corresponds to S–CH–S groups, which can demonstrate the click reaction between thiol and alkyne groups.

**Figure 4:**
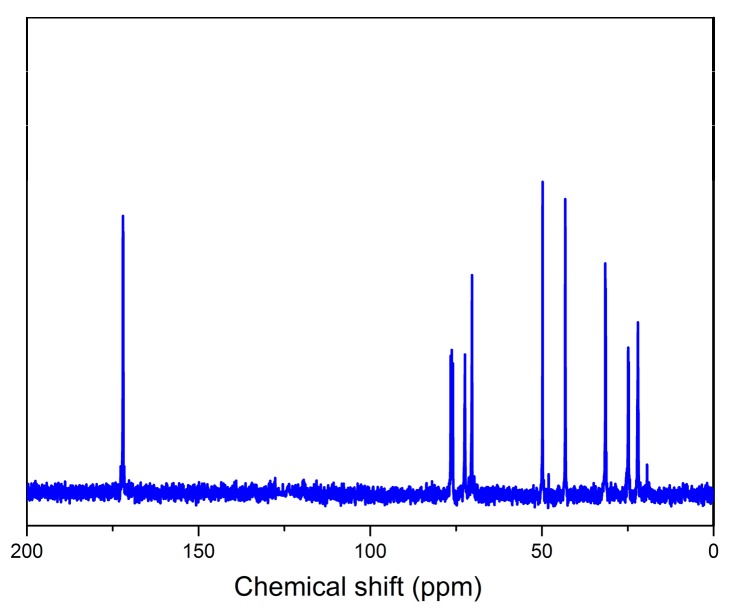
^13^C nuclear magnetic resonance (NMR) spectrum of HBP cleaved from the TiO_2_-HBP surface.

^13^C NMR spectroscopy was used to further verify the chemical structure of the grafted hyperbranched polymers (HBP), and the results are shown in Figure 4. The peaks at 72.4 and 76.7 ppm correspond to the chemical shifts of the carbon atoms in the unreacted alkyne groups (H–C**≡**C). Peaks at 171.8 and 70.2 ppm are attributed to the carbon atoms in the O–C(O)–CH_2_ and O–CH_2_–CH_2_–CH_2_–CH groups, respectively. In addition, the signals at 49.8 and 42.8 ppm can be assigned to the carbon atoms in the S–CH–S and CH–CH_2_–CH_2_–CH_2_–O groups, indicating that the alkyne groups react with thiol groups to form saturated bonds via click reaction. ^1^H NMR and ^13^C NMR analyses results confirm the chemical structure of hyperbranched polymers on the TiO_2_-HBP surface, which is the product of the click reaction between thiol groups on TiO_2_ and hyperbranched monomers.

**Figure 5:**
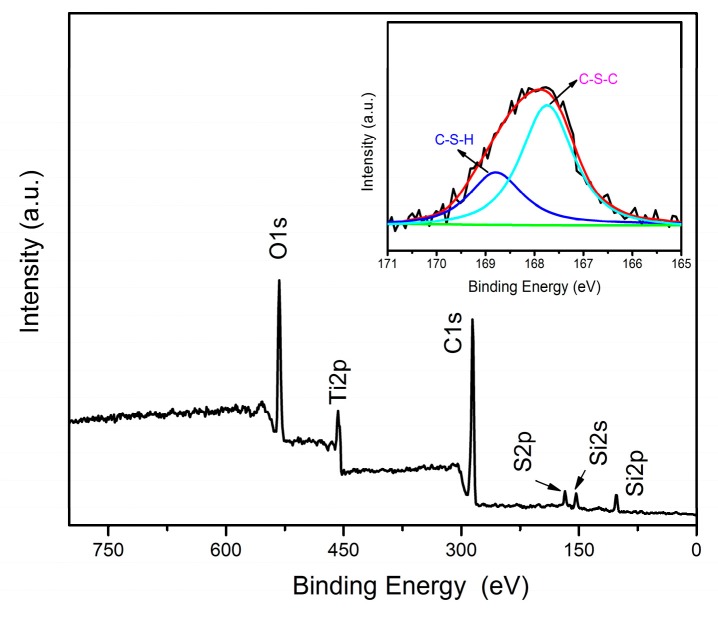
X-ray photoelectron spectroscopy (XPS) spectrum and S2p high-resolution spectrum of TiO_2_-HBP.

The X-ray photoelectron spectroscopy was used to identify the specific information about the elemental compositions of TiO_2_-HBP, and the results are shown in Figure 5. It can be found that the spectrum of TiO_2_-HBP shows C1s at 285.1 eV, O1s at 532.0 eV, Ti2p at 456.8 eV, Si2p at 102.1, Si2s at 151.8 eV, and S2p at 167.8 eV. The Si element originates from KH590 on the TiO_2_ surface. Furthermore, the S element may originate from KH590 or HBP. The XPS S2p spectrum was fitted into two peak components with binding energies at 167.6 eV and 168.7 eV, which are attributed to C–S–C and C–S–H. The presence of C–S–C demonstrates that the covalent bonds were formed between the TiO_2_ nanoparticles and HBP. The XPS results further confirm that the hyperbranched polymers were successfully grafted onto the TiO_2_ surface via thiol-yne click reaction.

**Figure 6:**
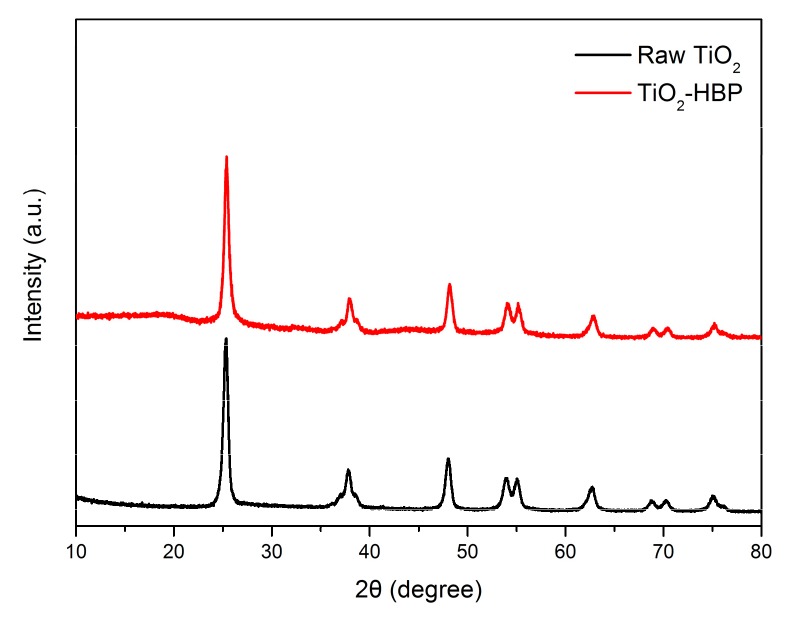
X-ray diffraction (XRD) spectra of raw TiO_2_ and TiO_2_-HBP.

The XRD technique was used to investigate the structural changes of TiO_2_ nanoparticles before and after grafting of hyperbranched polymers. Figure 6 shows the XRD spectra of raw TiO_2_ and TiO_2_-HBP. The raw TiO_2_ nanoparticles exhibited several sharp diffraction peaks at 2θ =25.3°, 37.9°, 48.3°, 54.1°, 55.1°, 62.8°, 68.8°, 70.3° and 75.1°, which are typical characteristics of anatase TiO_2_. Compared to the raw TiO_2_ nanoparticles, TiO_2_-HBP showed a broad hollow at 2θ = 17.9°, which can be assigned to the amorphous state of grafting hyperbranched polymers. The spectrum of TiO_2_-HBP also showed sharp diffraction peaks at the same position as raw TiO_2_ nanoparticles, indicating that the grafting of HBP did not change the crystalline structure of TiO_2_ nanoparticles significantly.

**Figure 7:**
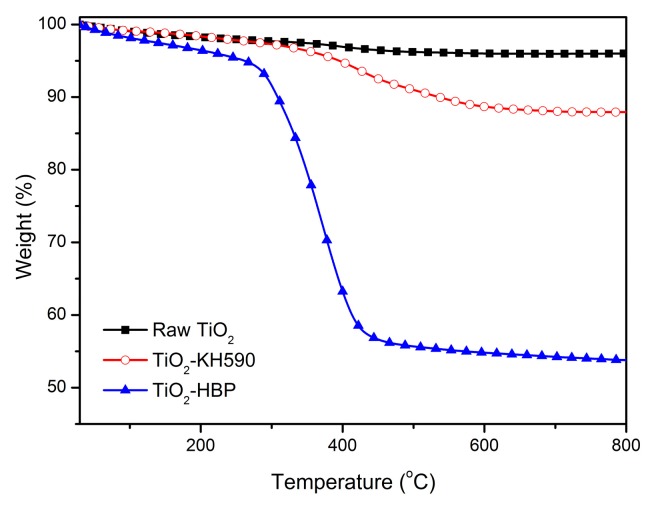
Thermogravimetry analysis (TGA) curves of raw TiO_2_, TiO_2_-KH590, and TiO_2_-HBP.

In order to verify the grafting content of hyperbranched polymers on the TiO_2_ surface, a thermogravimetric analysis (TGA) was performed on the raw TiO_2_ and surface functionalized TiO_2_, as shown in Figure 7. It can be found that a weight loss of 3.9% for the raw TiO_2_, which is attributed to adsorbed water or hydroxyl groups. TiO_2_-KH590 showed a weight loss of 12.1% at 800 °C, which is mainly due to the loss of KH590 on the TiO_2_ surface. For the TiO_2_-HBP, a major decomposition between 320 °C to 450 °C was found, which can be attributed to the decomposition of the hyperbranched polymers and KH590. As shown in Figure 7, weight loss of TiO_2_-HBP at 800 °C was about 46.7%. Therefore, the hyperbranched polymer content of TiO_2_-HBP was about 34.6% as estimated by thermogravimetry analysis.

### 3.2. Effect of TiO_2_ on the Mechanical and Thermal Properties of Nanocomposite Coatings

The tensile strength and elongation at break of nanocomposite coatings are summarized in Table 1. It is apparent that all of nanocomposite coatings containing TiO_2_ exhibited higher tensile strength and elongation at break than those of neat polyurethane coating. Moreover, the TiO_2_-HBP nanoparticles had a larger effect on the improvement of the mechanical properties of nanocomposite coatings, compared to the raw TiO_2_ nanoparticles. When the TiO_2_-HBP content was about 6 wt%, the tensile strength and elongation at break of TiO_2_-HBP/PU-6 nanocomposite coating increased to 16.98 MPa and 384%, respectively. Meanwhile, the relatively smaller improvement in tensile strength and elongation at break of about 12.93 MPa and 336% was shown in the nanocomposite coating containing the same content of the raw TiO_2_. The remarkably enhanced tensile strength and elongation at break can be attributed to less agglomeration of TiO_2_-HBP in the matrix and strong interaction between TiO_2_-HBP and the polyurethane matrix. These arise from the wrapping of TiO_2_-HBP with hyperbranched polymers, leading to the efficient transfer of the external force from the polyurethane matrix to TiO_2_-HBP nanoparticles. However, as the content of nanoparticles was higher than 6 wt%, the mechanical properties for two nanocomposite systems exhibited somewhat of a decrease, which may relate to an increasing susceptibility of agglomeration of nanoparticles with increasing nanoparticles content.

**Figure 8:**
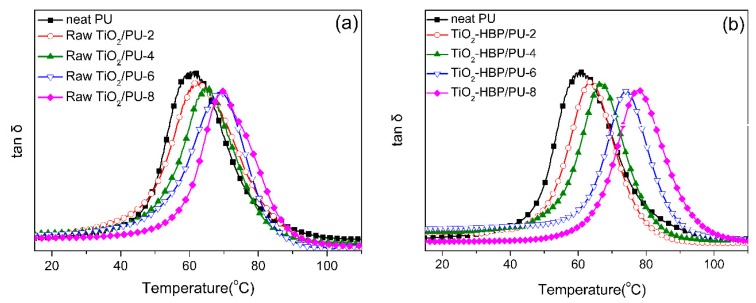
The loss factor curves of (**a**) raw TiO_2_/ polyurethane (PU) nanocomposite coatings and (**b**) TiO_2_-HBP/PU nanocomposite coatings.

The effect of TiO_2_ nanoparticles on thermal properties of coatings was studied by means of DMTA. Figure 8 shows the loss factor (tan δ) curves of neat polyurethane, raw TiO_2_/PU, and TiO_2_-HBP/PU nanocomposite coatings, and the peak position of tan δ is defined as the glass transition temperature (*T*_g_). It was clearly found that the introduction of TiO_2_ nanoparticles in the polyurethane matrix can lead to the increase of *T*_g_ values. Moreover, the increase of *T*_g_ values was more prominent in the TiO_2_-HBP/PU nanocomposite coatings, compared to those in the raw TiO_2_/PU nanocomposite coatings. The reason may be that a layer of hyperbranched polymers was formed on the TiO_2_-HBP surface after the click reaction. The presence of hyperbranched polymers on the TiO_2_ surface lead to an increase in the interaction between nanoparticles and the matrix, which limited the mobility of the matrix backbone. In addition, the TiO_2_-HBP nanoparticles exhibited better dispersibility in the polyurethane matrix and less tendency to form aggregates than the raw TiO_2_ nanoparticles. As a result, the TiO_2_-HBP nanoparticles showed a better enhancement effect on the T_g_ of nanocomposite coatings.

### 3.3. Surface Structure and Properties of TiO_2_/PU Nanocomposite Coatings

**Figure 9:**
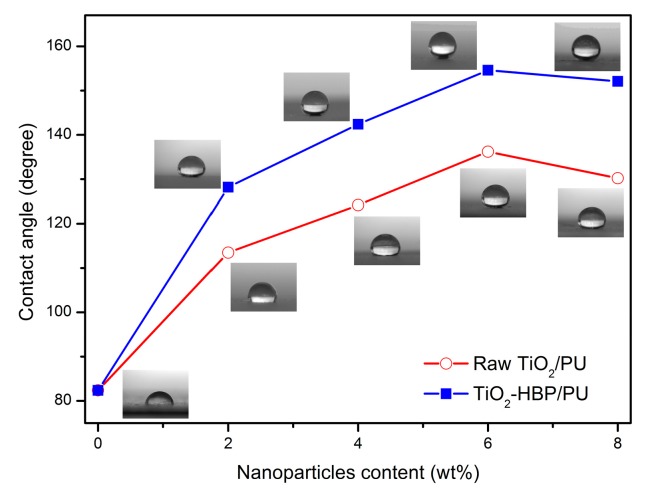
The water contact angle (CA) of nanocomposite coatings.

The introduction of TiO_2_ nanoparticles into polyurethane coating was expected to generate the superhydrophobicity, which can extend its application range. The superhydrophobicity of the nanocomposite coatings was evaluated by measuring the water contact angle (CA) values, and the results are shown in Figure 9. As shown in Figure 9, the water CA of neat polyurethane coating was only by about 82.4°. Meanwhile, the water CA of raw TiO_2_/PU nanocomposite coatings increased with increasing the raw TiO_2_ content. The water CA of TiO_2_-HBP/PU nanocomposite coatings also increased with increasing the TiO_2_-HBP content, and the enhancement effect was more notable than the raw TiO_2_/PU nanocomposite coatings. When the TiO_2_-HBP content was 6 wt%, the water CA of TiO_2_-HBP/PU-6 nanocomposite coating reached the largest value of 154.8°, meaning the formation of a superhydrophobic surface. This may be related to the low surface energy of hyperbranched polymers grafted on TiO_2_ surface and a larger surface roughness of TiO_2_-HBP/PU-6 nanocomposite coating. However, as the content of TiO_2_-HBP was more than 6 wt%, the water CA of nanocomposite coatings showed somewhat of a decrease. This is because a large amount of TiO_2_-HBP tends to aggregate, resulting in the decrease of surface roughness and the water CA value.

**Figure 10:**
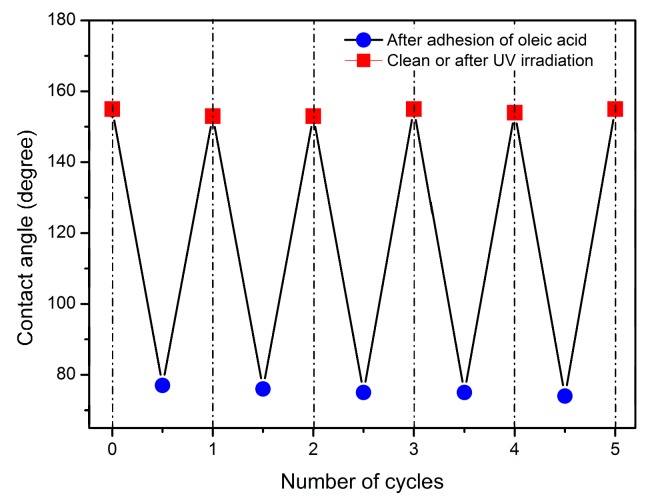
The water CA on TiO_2_-HBP/PU-6 nanocomposite coating in five cycles of oleic acid adhesion and UV irradiation.

Usually, the superhydrophobicity of conventional superhydrophobic coatings was hardly recovered without cleaning when the surface was contaminated with organic dirt. The addition of TiO_2_ nanoparticles can endow the coating with photocatalytic depollution performance. In this work, oleic acid was used as a representative contaminant, and Figure 10 exhibits the water CA changes on the TiO_2_-HBP/PU-6 nanocomposite coating in the five consecutive cycles of the oleic acid adhesion and degradation. After the adhesion of oleic acid, the water CA of TiO_2_-HBP/PU-6 nanocomposite coatings decreased from 154.8° to 77.2°, and the surface was converted to a hydrophilic state owing to a hydrophilic -COOH group on the oleic acid. When the TiO_2_-HBP/PU-6 nanocomposite coating is irradiated by UV light for 8 h, the water CA returns to 153.5°. Even after five cycles of oleic acid adhesion and UV irradiation, the surface of TiO_2_-HBP/PU-6 nanocomposite coating could still recover its superhydrophobic state. This is related to the strong photocatalytic oxidation power of TiO_2_, which can effectively decompose the oleic acid adsorbed on the surface. The experimental results demonstrate that the TiO_2_-HBP/PU nanocomposite coatings exhibited very good photocatalytic depollution performance.

**Figure 11:**
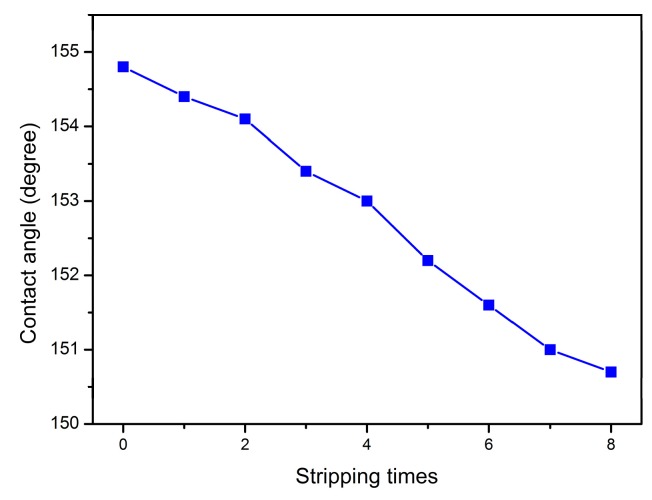
Relationship between the water CA of TiO-HBP/PU-6 nanocomposite coating and the number of stripping tests.

The stripping resistance of superhydrophobic coating is important for its industrial applications, which can be evaluated by using a bonding-stripping test. As shown in Figure 11, the water CA of TiO_2_-HBP/PU-6 nanocomposite coating was still over 150° after being stripped by test glues for 8 times, indicating that the nanocomposite coating retained its superhydrophobicity. The high stripping resistance of TiO_2_-HBP/PU-6 nanocomposite coating mainly originates from the strong interaction between TiO_2_-HBP and the polyurethane matrix.

**Figure 12:**
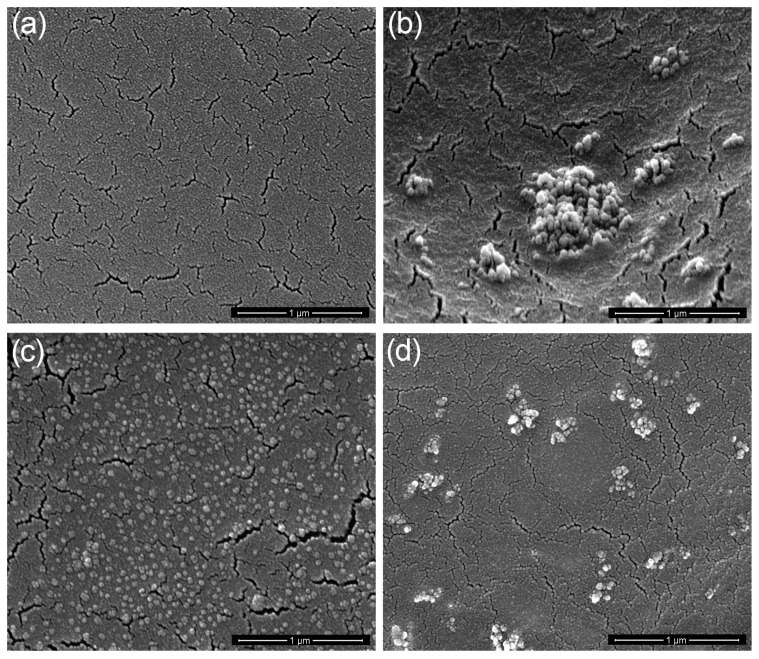
Scanning electron microscopy (SEM) images of (**a**) neat polyurethane coating, (**b**) raw TiO_2_/PU-6 nanocomposite coating, (**c**) TiO_2_-HBP/PU-6 nanocomposite coating, and (**d**) TiO_2_-HBP/PU-8 nanocomposite coating.

The scanning electron microscopy (SEM) was adequately performed to observe the morphology of the TiO_2_/PU nanocomposite coatings, and the SEM images of samples are shown in Figure 12. As shown in Figure 12a, it is clearly seen that the SEM image of neat polyurethane coating formed a relatively smooth surface. Meanwhile, the surface morphology of raw TiO_2_/PU-6 nanocomposite coating (Figure 12b) exhibits an obvious biphase character and numerous micro-scaled agglomerates of TiO_2_ nanoparticles, resulting from the high surface energy of raw TiO_2_ nanoparticles. On the contrary, the dispersion of TiO_2_-HBP on the surface morphology of TiO_2_-HBP/PU-6 nanocomposite coating (Figure 12c) was more uniform, which may relate to the presence of hyperbranched polymer chains on the TiO_2_ surface. The introduction of HBP on the TiO_2_ surface can significantly weaken the hydrogen bonding interactions between nanoparticles and cause a significant decrease in agglomeration. In addition, TiO_2_-HBP/PU-6 nanocomposite coating exhibited a lotus leaf-like microstructure. This special structure can increase the surface roughness and endow the nanocomposite coatings with better hydrophobicity. In Figure 12d, there are many irregular aggregates on the surface of TiO_2_-HBP/PU-8 nanocomposite coating because of an excessive amount of TiO_2_-HBP which self-aggregates and thus decreases the roughness of the surface and mechanical properties of coating.

## 4. Conclusions

In order to modify the surface of TiO_2_ nanoparticles and increase the interaction between nanoparticles and polymer matrix, the hyperbranched polymers were chemically grafted onto the surface of TiO_2_ via thiol-yne click chemistry. Experimental results show that the introduction of HBP improved the TiO_2_ dispersion and imparted lotus leaf-like microstructures at the nanocomposite coatings surface, resulting in the increase of the surface roughness and accordingly an increase of the water CA value. As the TiO_2_-HBP content is about 6 wt%, the water CA of nanocomposite coatings reached a maximum value of 154.8°, indicating the formation of a superhydrophobic surface. The mechanical and thermal properties of the nanocomposite coatings were improved with an increasing TiO_2_-HBP content. The resulted nanocomposite coating containing 6 wt% of TiO_2_-HBP showed a high photocatalytic depollution performance and stripping resistance, retaining its superhydrophobicity after being immersed in oleic acid or stripped by test glues for 8 times, which makes this nanocomposite coating a promising candidate for a wide range of superhydrophobic applications.

## Figures and Tables

**Table 1:** Tensile strength and elongation at break of the nanocomposite coatings.

Sample	TiO_2_ Content (wt%)	Tensile Strength (MPa)	Elongation at Break (%)
Neat PU	0	10.58	278
Raw TiO_2_/PU-2	2	11.15	313
Raw TiO_2_/PU-4	4	11.38	325
Raw TiO_2_/PU-6	6	12.93	336
Raw TiO_2_/PU-8	8	12.45	313
TiO_2_-HBP/PU-2	2	12.53	348
TiO_2_-HBP/PU-4	4	14.95	368
TiO_2_-HBP/PU-6	6	16.98	384
TiO_2_-HBP/PU-8	8	15.48	370
